# Imprinted Polydimethylsiloxane-Graphene Oxide Composite Receptor for the Biomimetic Thermal Sensing of *Escherichia coli*

**DOI:** 10.1021/acssensors.2c00215

**Published:** 2022-05-10

**Authors:** Rocio Arreguin-Campos, Kasper Eersels, Renato Rogosic, Thomas J. Cleij, Hanne Diliën, Bart van Grinsven

**Affiliations:** Sensor Engineering Department, Faculty of Science and Engineering, Maastricht University, P.O. Box 616, 6200 MD Maastricht, The Netherlands

**Keywords:** Biomimetic sensing, imprinted polymers, food safety, cell imprinting, graphene oxide

## Abstract

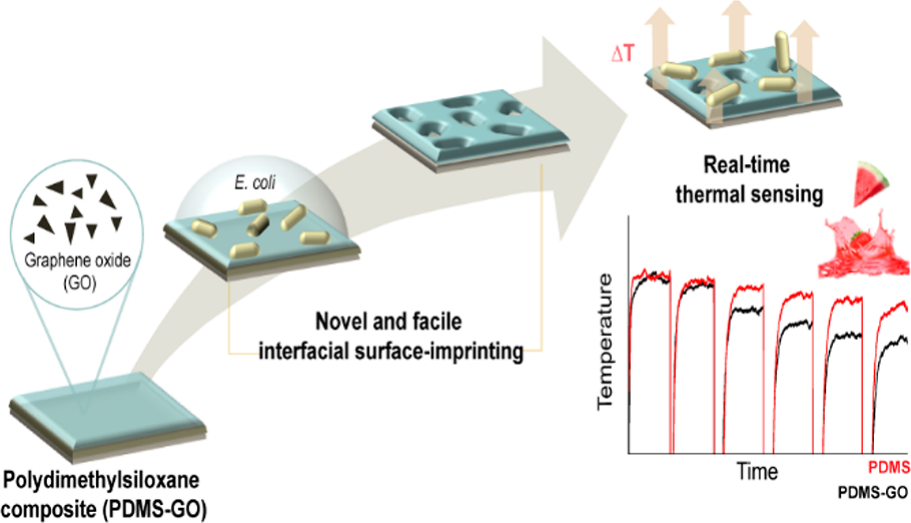

This work presents an imprinted polymer-based thermal biomimetic sensor for the detection of *Escherichia coli*. A novel and facile bacteria imprinting protocol for polydimethylsiloxane (PDMS) films was investigated, and these receptor layers were functionalized with graphene oxide (GO) in order to improve the overall sensitivity of the sensor. Upon the recognition and binding of the target to the densely imprinted polymers, a concentration-dependent measurable change in temperature was observed. The limit of detection attained for the sensor employing PDMS-GO imprints was 80 ± 10 CFU/mL, a full order lower than neat PDMS imprints (670 ± 140 CFU/mL), illustrating the beneficial effect of the dopant on the thermo-dynamical properties of the interfacial layer. A parallel benchmarking of the thermal sensor with a commercial impedance analyzer was performed in order to prove the possibility of using the developed PDMS-GO receptors with multiple readout platforms. Moreover, *S. aureus*, *C. sakazakii* and an additional *E. coli* strain were employed as analogue species for the assessment of the selectivity of the device. Finally, because of the potential that this biomimetic platform possesses as a low-cost, rapid, and on-site tool for monitoring *E. coli* contamination in food safety applications, spiked fruit juice was analyzed as a real sample. Reproducible and sensitive results fulfill the limit requirements of the applicable European microbiological regulation.

Bacteria are ubiquitous microorganisms. While the majority of them are involved in beneficial interactions with the environment, animals, and humans, certain microbes possess the potential of causing infectious diseases. *Escherichia coli*, for instance, is a bacterium typically found in the human intestinal tract.^1^ These microorganisms, which can be transmitted via multiple pathways such as water, soil, and food, are employed as environmental faecal indicators, suggesting the presence of harmful bacteria.^2^ Some *Escherichia coli* strains have been identified multiple times as the origin of foodborne illness outbreaks with global repercussions at public health and economical levels.^3^ In order to avoid this, food processors employ routine bacteria detection methods such as plate counting and molecular-based technologies (e.g., immunoassays)^4^ to monitor the microbial contents of their products qualitatively or quantitatively. Although these procedures are selective and sensitive, they can be time-consuming, laborious, and in some cases, costly. Therefore, a lot of research efforts in the last decades have been focused on the development of alternative detection technologies that that allow fast, cost-effective, and accurate detection of bacteria along the food supply chain.^5^

Biosensors for bacteria detection have been developed in diverse fields where rapid, on-site testing is needed, such as medical diagnosis or environmental monitoring.^6,7^ In food safety, research suggests that these devices possess the potential to overcome inherent challenges of foodstuff analysis, namely, complex matrices and attaining sensitivities that comply with the applicable microbiological criteria.^8,9^ Several commercial platforms for the detection of low-molecular-weight contaminants in food products are already on the market. Nonetheless, the usage of biological receptors in sensing platforms also holds some limitations including fragility, the requirement of carefully regulated conditions (pH, ionic strength, temperature, etc.), and limited shelf life.^10^ In order to fill in this gap, imprinted polymers as biomimetic alternatives have been recognized for their chemical stability and desirable affinities. The possibility of combining these synthetic receptors with a wide variety of transducing technologies (optical,^11^ electrochemical,^12^ mass-sensitive,^13^ thermal^14^) has made possible already their use in the detection of food contaminants.

The authors of this paper have reported previously on the development of the heat-transfer method (HTM),^15^ a versatile inexpensive thermal readout technology that has received increasing attention in the past few years. Combining HTM with imprinted polymers has proven to be a particularly valuable approach for the construction of sensors for the detection of a wide range of targets, including small molecules,^16^ human cells,^17^ and bacteria.^18^ The fundamentals of the HTM rely on the measurement of the changes in the thermodynamic properties of the polymer that derive when the analyte binds to the synthetic receptor.

When the HTM was investigated for bacteria detection, the sensitivity of the device was identified as an improvement area.^18^ The limit of detection for *Escherichia coli* (10^4^ CFU/mL) hindered the introduction of the sensor to diverse applications including its use in food safety management. This sensitivity is directly influenced by the two components of the device: the synthetic recognition element and thermal readout platform. In order to attain a reproducible and appreciable binding behavior, the synthetic receptor has to be prepared considering a variety of experimental parameters such as material selection (functional monomers and ratios) as well as the polymer cell-imprinting technique.^19^ In order to achieve the creation of cavities on the surface, specialized protocols such as the use of template stamps in microcontact imprinting are required.^20^ This not only introduces batch to batch variations but also hampers the scalability of imprint preparation. Moreover, on the transducer platform side, polymers are known for possessing low thermal conductivity (usually lower than 0.5 W/mK),^21,22^ which impacts in the noise of the device when translating the signal derived from the recognition event.

A couple of years ago, a modification of the HTM consisting on the implementation of a planar meander element was reported,^23^ resulting in a lower noise level in the device and, therefore, a significant decrease in the detection limit of the sensor down to 100 CFU/mL. Although this improvement pushed forward the applicability of the sensor, the thermal readout platform was modified in terms of flow cell design and components, compromising the simplicity and cost-effectiveness of the original device. Similarly, stringently controlled advanced polymer imprinting techniques are more complex and require additional instrumentation for upscaling polymer synthesis.

In this work, we propose a sensor design that aims to enhance sensitivity while maintaining the original simple thermal readout platform. This approach targets the aforementioned challenges of synthetic receptor preparation in terms of material selection and imprinting protocol. In order to avoid ab initio laborious imprinted polymer synthesis, the commercial available elastomer polydimethylsiloxane (PDMS), known for its moldability and chemical and mechanical robustness,^24^ is proposed for its use as recognition element in the HTM biomimetic platform. This material has been cell and bacteria-imprinted employing microcontact^25^ and roll-to-toll techniques,^26^ exhibiting the ability to recognize and sort these targets based on both morphology and chemical functionality. Hereby, we investigate a novel and simple surface imprinting protocol for PDMS that consists in the free assembly of the microorganism onto the surface of the polymer without the aid of a stamp. This approach enables for the first time scalability in the preparation of the receptor layers. Furthermore, in order to address the inherent low thermal conductivity of the synthetic receptor we suggest the use of a functional additive in order to improve the response of the HTM transducer.

Graphene oxide (GO) has been widely researched as filler for polymers. It has been observed that even a small loading of this carbon material has the ability of transferring its outstanding physicochemical properties to the composite.^27^ This attribute of GO has been already explored in imprinted polymers in combination with electrochemical readouts.^28^ In this work, we propose the use of graphene oxide flakes as filler for imprinted PDMS layers with the aim of obtaining a material with increased thermal conductivity, investigating the impact on the overall sensitivity of the sensor. Moreover, in order to benchmark with a commercial transducing platform, these results are compared in parallel with an impedance analyzer. Finally, the proposed sensor is validated in fruit juice and correlated to the applicable legal limits established by the European Commission in order to explore the application of the proposed sensor in food safety.

## Materials and Methods

### Chemicals and Reagents

Lysogeny broth (LB), CASO broth, safranin, sodium dodecyl sulfate (SDS), phosphate buffer saline (PBS), and anhydrous tetrahydrofuran (THF) were obtained from Merck (Diegem, Belgium). Ethanol 70% was purchased from VWR international, and BacLight bacteria fluorescent stain from Fisher Emergo (Landsmeer, Netherlands). All reagents were used as received and had a minimum purity of 99.9%. *E. coli* (ATCC 8739), *E. coli* (ATCC 23716), *Cronobacter sakazakii* (ATCC 29544), and *Staphylococcus aureus* (ATCC 6538) strains from DSM-Z (Braunschweig, Germany). Polydimethylsiloxane Sylgard 184 elastomer kit was purchased from Mavom N.V. (Schelle, Belgium). Graphene oxide (GO) flakes synthesized following an improved Hummer’s method^29^ was provided from Aachen-Maastricht Institute for Biobased Materials (AMIBM), The Netherlands. All aqueous solutions were prepared with deionized water with a resistivity of 18.1 MΩ cm^–1^.

### Bacteria Culturing and Preparation of Bacteria Suspensions

Broths were prepared according to the standard protocols. Initially, 25 mL of medium were inoculated with a single colony of bacteria and gently shaken at 120 rpm overnight at 37 °C. Subsequently, 0.5 mL of the cultures was diluted in 4.5 mL of fresh broth and left for further growth for 2 h. Bacteria concentrations were calculated by measuring the OD600. The cultures were centrifuged at 0.9 RCF for 5 min, and the obtained pellets were resuspended in PBS. This washing procedure was repeated once, and lastly, the bacteria were diluted with sterile PBS to obtain the desired concentrations.

### Preparation of Polydimethylsiloxane and Polydimethylsiloxane-Graphene Oxide Composite Stock Solutions

Graphene oxide flakes were dispersed into polydimethylsiloxane base resin employing a Fisherbrand sonic dismembrator with a probe of 2 mm diameter. In order to avoid heat generation, the PDMS was kept in an ice-bath during the sonication process. Subsequently, the base containing GO was mixed with the curing agent following the manufacturer’s suggested ratio (10:1 (w/w)). The viscous mixture was employed for preparing a stock solution of 10% PDMS in tetrahydrofuran (w/w). A stock solution of neat PDMS was prepared following the same procedure except for the addition of GO.

### Interfacial Polymer-Imprinting

Microscope glass slides and 1 cm^2^ aluminum chips were spin-coated for 60 s at 5000 rpm with 150 μL of the prepared PDMS and PDMS-GO stock solutions. A precuring treatment for the resin was performed on the substrates at 65 °C for 10 min. Subsequently, 250 μL of template bacteria (*E. coli* ATCC 8739) solution (1× 10^8^ CFU/mL) was applied onto the surface of the precured films and left for sedimentation for 20 min at room temperature. While maintaining the bacteria solutions on the surface of polymers, the substrates were placed in the oven at 65 °C for 3 h in order to achieve full curing of the PDMS. Films were finally washed with deionized water in order to remove residual salts from PBS buffer followed by SDS 3% to detach the template from the PDMS leaving behind the imprint cavities.

### Optical Characterization of the Polymer Imprint’s Surfaces

Bright-field microscopy was performed on a LEICA DM 750 optical microscope. ImageJ 1.44O (National Institute of Health, Bethesda, MA) was employed to calculate the average surface coverage of cell imprints on the polymeric layers. The number of cell imprints per area unit was determined on the basis of the individual counts of three different batch samples and three locations on each imprint. In order to facilitate the visualization of the bacteria, safranin was employed as staining solution.

Fluorescence microscopy was performed on an Olympus BX53 microscope. With the aim of visually confirming the rebinding of the targeted bacteria to the prepared imprints, *E. coli* was stained with fluorescent dye according to the standard protocol. Imprinted films were exposed to a solution of 1 × 10^8^ stained bacteria mL^–1^ for 20 min in order to allow recognition of the target. After this time, the films were rinsed with PBS in order to remove nonbound bacteria from the surface. The films were then observed under the microscope.

Scanning electron microscopy was carried out at DSM, Geleen, Netherlands on a Thermo Fisher Scientific FEI Teneo at 2.0 eV, using an iridium coating. The prepared imprinted polymers were observed in order to confirm the presence of the cavities on the surface and to analyze their morphology.

### Heat Transfer Method, Impedance Measurements, and Setup

The setup for the HTM device has been described earlier.^15,30^ Briefly, the surface-imprinted chips were placed backside onto a copper block that performs as heat sink. The block is then coupled to a PMMA (poly methyl methacrylate) flow cell by sealing the two pieces with an O-ring to avoid leakage. The contact area of the imprint is determined by the diameter of this ring (28 mm^2^), and the volume of the flow cell is 110 μL, which are introduced to the system using a tubing system via an automated syringe pump. The temperature of the heating block (T1 = 37 °C) is controlled by modifying the voltage over the power resistor (Farnell, Utrecht, The Netherlands) using a proportional-integral-derivative (PID) software-based controller (Labview, National Instruments, Austin, TX). The settings employed have been optimized in previous work (P = 10, I = 8, D = 0). T1 and the temperature of the chamber (T2) were monitored by K-type thermocouples (TC direct) placed in the copper block and at 1 mm above the chip, respectively.

Impedance was measured with a MFIA impedance analyzer from Zurich Instruments (Zurich, Switzerland). For this purpose, a gold wire of 0.5 mm diameter was adapted into the flow cell as electrode at the same position from the bottom of the chamber as the thermocouple in the opposite side. Continuous frequency sweeps of 200 points were taken between 100 and 10000 Hz at a test signal of 300 mV. Before each experiment, PBS is introduced into the flow cell, and the system is allowed to stabilize. After this stabilization period, 2 mL of the desired bacteria solution is injected at a controlled flow rate of 2 mL/min. The stabilization time employed for bacteria in the system was 20 min, and afterward, a solution of SDS (3%) followed by PBS are flushed into the flow cell at the previously mentioned flow rate with the purpose of removing the bacteria from the polymer layers. The HTM setup monitors the temperature and thermal resistance (R_th_) and the electrode the impedance at the solid–liquid interface simultaneously. Dose–response curves for HTM were obtained from temperature data as reported previously for chemo-sensing employing the HTM.^31,32^ As for impedance, curves were obtained from the absolute impedance values at a single frequency at which the corresponding phase angle is between 40 and 50 degrees, focusing on the double layer represented by an “R-C” circuit.

Calculations derived from HTM and impedance transducers (effect sizes and limits of detection) were made employing three independent measurements of surface-imprinted chips.

### Food Sample Analysis

Watermelon-strawberry juice (not heat-treated) was bought from Albert Heijn supermarket (Maastricht, The Netherlands) and used as received. After being tested for the absence of microorganisms, it was spiked with *Escherichia coli* to obtain the desired concentrations. No additional sample preparation was performed.

## Results and Discussion

### PDMS Interfacial Imprinting

**Figure 1:**
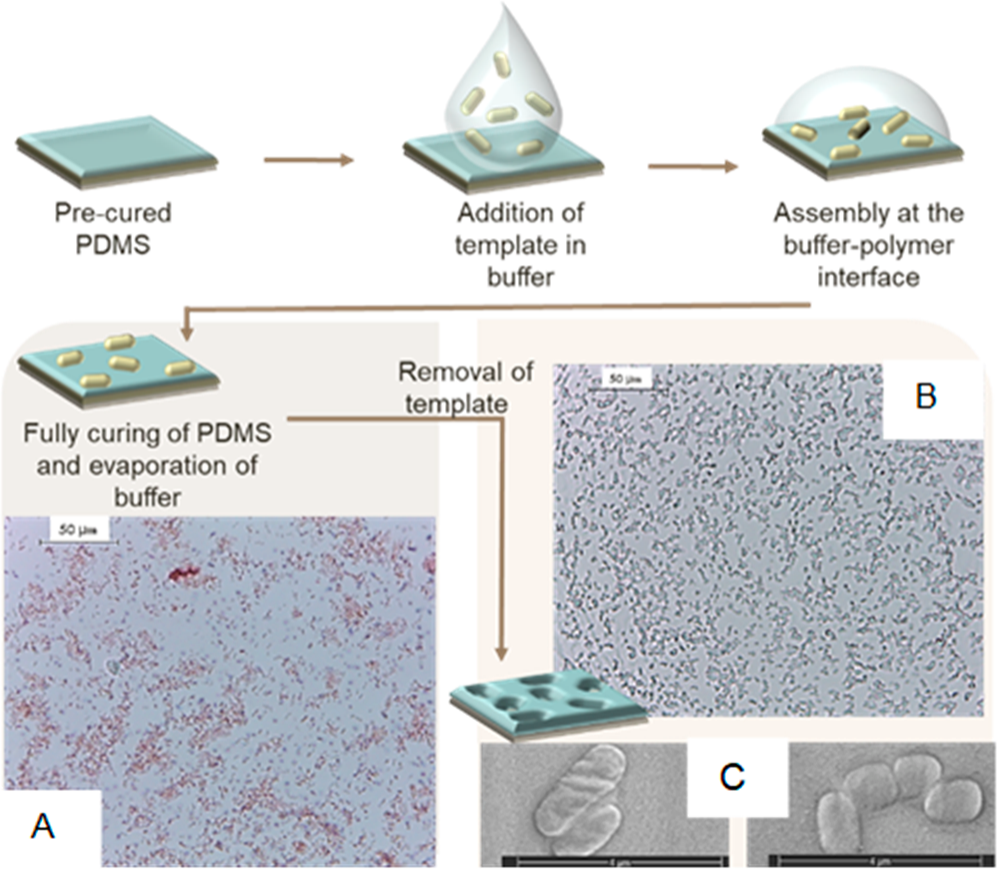
Schematic representation of the interfacial surface-imprinting process of PDMS. (A) Brigthfield microscopy of *E. coli* safranin-stained on imprinted polymer after curing. (B) Brigthfield microscopy of empty bacteria cavities on polymer. (C) Scanning electron microscopy of bacteria imprints after the removal of the template.

Visual assessment of the surface-imprinted polymers (SIPs) was performed using Brigthfield microscopy with the aim of confirming the presence of bacteria cavities on the PDMS layers. Figure 1A depicts the imprinted polymer right after its preparation, where the presence of safranin-stained bacteria on the films can be observed. The analyte presents a heterogeneous distribution, and the notorious agglomeration of *E. coli* can be highlighted because of the imprinting technique employed, in which the bacteria freely assembles onto the semicured PDMS. The optimal template concentration for the preparation of the imprints was determined by testing *E. coli* suspensions of 1 × 10^4^, 1 × 10^6^, 1 × 10^8^, and 1 × 10^9^ CFU/mL. Optical and quantitative results for these experiments are available in Supporting Information (Figure S1), from where it was assessed that a template concentration of 1 × 10^8^ CFU/mL is the most adequate for the imprinting. The density of template on the surface of the polymer was calculated as 21.7 ± 4.8%, an enhanced coverage when comparing with the values obtained for microcontact imprinting techniques (14.3 ± 1.8%) in previous studies.^18^ Removal of the bacteria was done by washing the imprinted layers with SDS 3% and with water to dissolve residual salts from the imprinting process. Figure 1B shows the surface of the material with empty cavities. The observed pockets on the PDMS match *E. coli* in shape and size, which was further confirmed with scanning electron microscopy, where the characteristic rod-shape morphology of *E. coli* (1–3 μm) was clearly identified (Figure 1C). The compilation of these results confirm that PDMS synthetic receptors for the analyte of interest *Escherichia coli* were successfully prepared via interfacial imprinting.

### Bacteria Detection Employing the Heat Transfer Method

The results in the previous chapter clearly illustrate that it is possibly to achieve a morphological imprint of the bacteria on the surface of the PDMS layer. In a next step, the receptor layers were prepared on aluminum chips in order to quantify the rebinding of the template in PBS using the HTM readout platform. In this study, we investigated for the first time the effect of a functional additive (GO) in the receptor layers coupled to HTM as a strategy to enhance the sensor’s signal without adding complexity to the readout method. For this purpose, neat PDMS as well as PDMS-GO (0.01%) composites were prepared. The selection of a small load of GO aims to maintain a fast and simple homogenization process for the filler. SEM imaging of the graphene oxide functionalized layers can be found in Supporting Information (Figure S2). Rebinding was tested on PDMS SIP, PDMS-GO SIP receptors and compared to the behavior of nonimprinted layers (NSIP).

**Figure 2:**
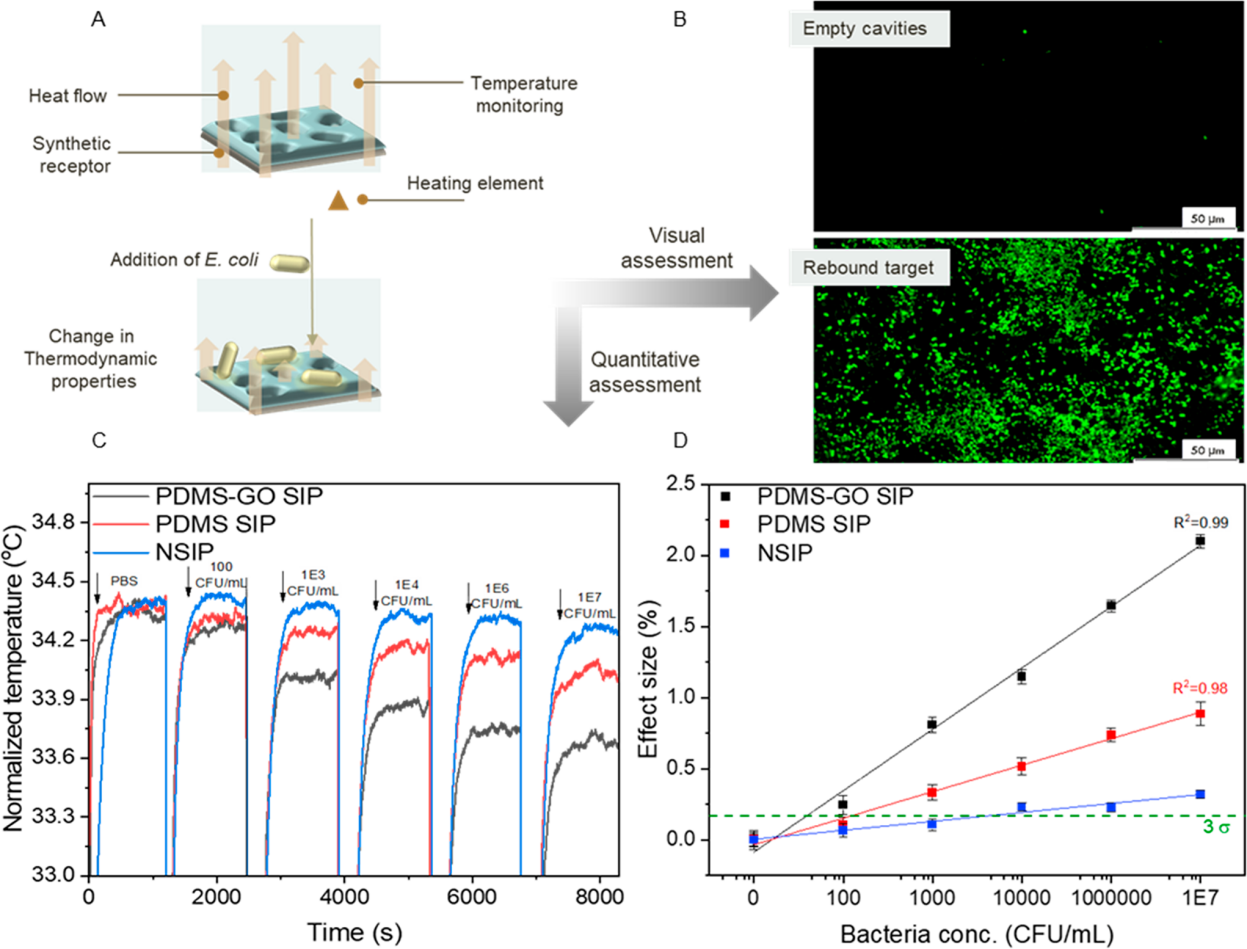
(A) Schematic representation of the thermal recognition of *E. coli*. (B) Fluorescence microscopy image visually depicting the rebinding of stained target to the empty cavities on the polymer (PDMS-GO SIP). After exposition to the target, the layers were rinsed with PBS in order to remove unbound cells. (C) Real-time temperature response of the sensor employing surface-imprinted PDMS SIP, PDMS-GO SIP receptors as well as the nonimprinted PDMS layers (NSIP). (D) Dose–response curve of the sensor employing the different receptor layers. The dashed line indicates the limit of detection, defined as three times the average of the error on the data. Error bars are calculated, making use of the noise of the signal of the sensor.

The experimental conditions for the HTM were kept constant in order to enable the direct comparison of the layers. The flow cell system was filled with phosphate buffer as blank and the temperature of the copper heating element was stabilized at 37 °C for 20 min. Subsequently, the receptors were exposed and incubated to an increasing concentration of *E. coli* suspensions in PBS in order to allow the target to bind to the surface (Figure 2A). Between each incubation step, the films were rinsed in situ with SDS (3%) and buffer with the purpose of removing all the bound cells from the previous exposition before adding the next concentration.

The results of these experiments are shown in Figure 2B, where the sensor’s real-time response can be observed for the different tested layers. In the case of PDMS SIP and PDMS-GO SIP, a clear diminishing of the flow cell’s inside temperature can be observed as the template concentration is increased, which is attributed to the augmentation in thermal resistance at the solid to liquid interface derived from the binding of the bacteria to the imprinted polymer (Figure 2A). This can be confirmed when analyzing PDMS without cavities, for which only a small temperature response is observed because of nonspecific interactions with the polymeric surface. Only when a high concentration of bacteria (10 000 CFU/mL) is infused into the system do we observe a significant response that barely exceeds the noise of the signal. Although both PDMS-based imprinted receptor layers exhibit this concentration-dependent trend of temperature change, it can be highlighted that the response obtained for the PDMS-GO receptors is more pronounced when compared with neat PDMS. This can be directly linked to the presence of the carbon functional additive, which has been reported to confer thermal properties to polymers when used (in flakes,^33^ fibers,^34^ nanoparticles,^35^ etc.) in composites. In order to link the temperature response obtained from the sensor to a visual confirmation of the template’s rebinding, the target was stained with a labeling reagent and incubated for 20 min on the surface of the imprinted PDMS-GO. Subsequently, the layers were rinsed with buffer to remove unbound cells. In Figure 2C, fluorescent microscope pictures of the synthetic receptor with and without the target depict the recognition event. Moreover, it can be highlighted that the heterogeneous and agglomerated distribution of rebound bacteria is in alignment with the observations made for the brigthfield images of the polymers after imprinting.

With the aim of determining the limit of detection (LoD) of the biomimetic sensor when employing the different PDMS receptor layers, dose–response curves were constructed with the real-time obtained temperature data. Mean values for each incubation steps were obtained from 300 s intervals after stabilization of the signal. Subsequently, effect sizes were calculated employing the average temperature^32^ for each target concentration (*t* = *c*) with respect to the temperature of the baseline (*t* = 0). The used formula was:
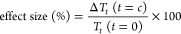


The effect size data of multiple experiments (in terms of chips and batches) was plotted against the normalized logarithmic bacteria concentrations and fit with OriginPro to an empirical linear function with the formula: *y* = *a*+ b*x (Figure 2D). The limits-of-detection were calculated as the lowest concentrations at which the effect size is higher than 3 times the averaged error collected for three data sets (green line, 3σ method). The LoD obtained for neat PDMS SIP was 670 ± 140 CFU/mL, which is in line with previously found values for bacteria-imprinted polyurethane layers.^36^ Furthermore, the limit for PDMS-GO SIP is 80 ± 10 CFU/mL, which confirms that the enhancement of the sensor’s signal due to the presence of graphene oxide as additive, leads to an improvement in the overall sensitivity of the device.

### Integration of Heat Transfer Method Sensor with Impedance Analyzer

The heat transfer method (HTM) platform has been proposed as a low-cost, rapid, and user-friendly technology for biomimetic sensing. Because of the research that is being performed on its optimization, it is of value to compare its performance with other transducers. In order to benchmark the PDMS-GO/HTM biomimetic sensor with a commercial readout technology, real-time experiments following the protocol employed for the HTM were performed using impedance spectroscopy. The representative curves corresponding to the simultaneous monitoring of these readout techniques against time can be found in Supporting Information (Figure S3). As observed for HTM, the sensor exhibited a concentration-dependent drop in impedance that can be attributed to the fact that *E. coli* possesses a negatively charged surface because of the presence of anionic groups on its outer membrane (e.g., carboxylates and phosphates).^37,38^ The drops in impedance and in temperature were employed for calculating the effect sizes and the limits of detection of the sensor by fitting the response of the platforms to an empirical bacterial growth equation integrated in the OriginPro software package with the formula: *y* = a*(*x*–*b*)^∧^*c*. LoD (3σ) and effect sizes are summarized in Table 1. These data illustrate that the impedimetric quantification of the template is 1 order of magnitude more sensitive when compared with HTM. This can be attributed to the fact the accumulation of charge of *E. coli* will create a larger relative effect size in impedance as compared with the thermal resistance signal. Furthermore, it can be noticed that the effect sizes for the thermal readout when integrating it with impedance are around 3 times lower compared with the results observed for the HTM by itself. This could be correlated to the aforementioned charge accumulation at the solid to liquid interface, which might create turbulence and hinder thermal transport from the receptor to the buffer liquid. This phenomenon leads to limits of detection that are higher and less reproducible and, therefore, suggests that whereas the PDMS-GO hereby presented are suitable for their use in both transducing platforms, better results can be expected when temperature and impedance are monitored separately.

**Table 1:** Simultaneous Integration of HTM and Impedance Transducers

	impedance		HTM	
batch^a^	LoD CFU/mL	effect size %	LoD CFU/mL	effect size %
1	26	6.2	120	0.67
2	30	7.3	700	0.44
3	30	15.1	321	0.64

a Imprints were prepared separately with different batches of PDMS-GO.

### Selectivity of the Receptor Layer

**Figure 3:**
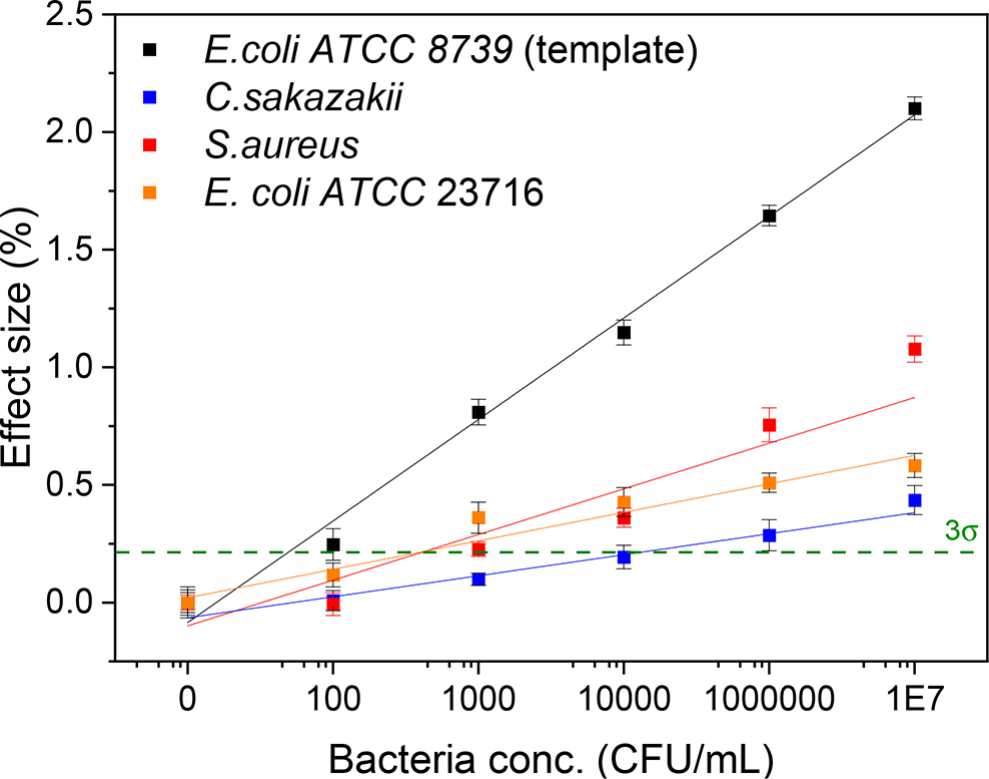
Effect size curves for selectivity of the PDMS-GO imprints. Receptors imprinted for a specific strain of *E. coli* were exposed to *S. aureus, C. sakazakii* and an additional *E. coli* strain. Bacteria concentrations employed were 0, 1 × 10^2^, 1 × 10^3^, 1 × 10^4^, 1 × 10^6^, and 1 × 10^7^ CFU/mL. Error bars are calculated making use of the noise of the signal of the sensor. The dashed line indicates the limit of detection, defined as 3 times the average of the error on the data.

*Staphylococcus aureus*, *Cronobacter sakazakii*, and an additional strain of *Escherichia coli* K-12 (ATCC 23716) were employed for assessing the selectivity of the PDMS-GO imprinted receptors. Increasing concentrations of these microorganisms were infused into the setup in separate experiments following the same conditions used for the targeted *E. coli* in order to compare the sensor’s response. The results for these tests are summarized in Figure 3, where it can be noticed that the device exhibited an effect size which is roughly twice for the *E. coli* strain that was employed as template in comparison to the nontargeted bacteria. This predominant effect can be attributed to the fundamentals of recognition of imprinted polymers, which rely on the chemical interaction of the analyte with the receptor and the geometrical matching of the cavities on the material.^39^ Nevertheless, a significant response is also obtained for *C. sakazakii* and *S. aureus*. This can be explained by the fact that *C. sakazakii* is structurally very similar to the template, while *S. aureus* is known to nonspecifically bind to a larger extent to PDMS than enterobacteriaceae because of electrostatic and hydrophobic forces.^40,41^ The signal does however only generate a significant increase at concentrations of 1E^4^ CFU/mL and higher. However, as *S. aureus* concentrations are known to be several orders of magnitude higher in food matrices than *E. coli*, the sensitivity limit of the sensor could make it difficult to distinguish between a low concentration of the target bacterium and a high concentration of other pathogens. To use the sensor platform in real-life situations, the selectivity will have to be tuned in terms of the desired application. The data on the experiment with the K-12 *E. coli* strain further emphasize this, as the surprisingly low degree of cross-selectivity observed would be interesting for some applications, whereas it would probably be less practical and harder to calibrate when the goal is to build a strain-independent *E. coli* sensor.

As cell recognition in surface imprinted polymers is determined predominantly by chemical interactions in addition to shape complementarity,^25^ it is possible to tune the selectivity of the system by introducing simple modifications to PDMS,^42−44^ in order to decrease or increase nonspecific interactions with other bacteria (e.g., hydrophobic or electrostatic).

### Food Sample

**Figure 4:**
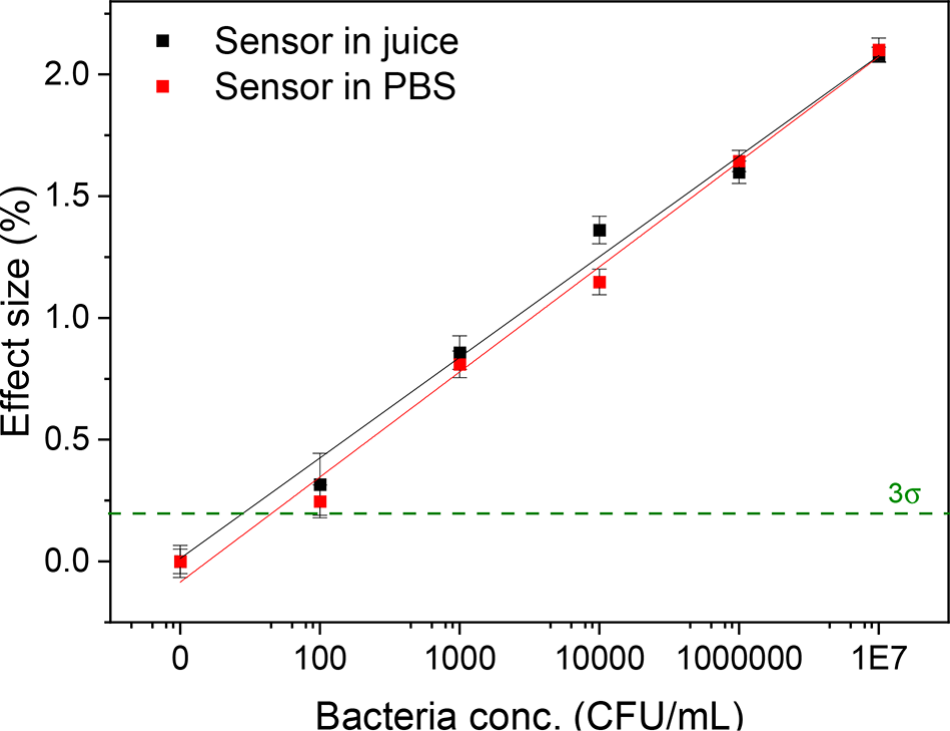
Effect size curve for detection of *E. coli* in juice. Bacteria concentrations employed were 0, 1 × 10^2^, 1 × 10^3^, 1 × 10^4^, 1 × 10^6^, and 1 × 10^7^ CFU/mL. Error bars are calculated making use of the noise of the signal of the sensor. The dashed line indicates the limit of detection, defined as 3 times the average of the error on the data

Spiked juice samples were analyzed with the PDMS-GO sensor and its performance was compared to the experiments in buffer summarized in a previous chapter. The corresponding effect size curves obtained from multiple experiments are shown in Figure 4, where the similarity of the device’s performance in both liquids can be observed. The limit of detection calculated for the food sample (70 ± 8 CFU/mL) is similar to the value obtained for PBS bacteria suspensions.

For this type of food sample (ready-to-drink fruit juices), the European Commission Regulation (EC) 1441/2007 on microbiological criteria for foodstuffs determines a legal limit of 100–1000 CFU/g for *Escherichia coli*.^45^ The results for the sensor hereby presented prove bacterial contamination in these type of juices as its linear range falls within these established values. These results confirm that the biomimetic sensing platform has undergone the necessary improvements in terms of sensitivity for its potential implementation into certain commercial food safety assessment processes. The limit of detection, enhanced by 2 orders of magnitude, derives from the synthetic receptor imprinting procedure and the use of graphene oxide as a functional additive.^18^

In Table 2, a summary of other relevant biomimetic platforms researched for *E. coli* in food samples in the past decade is presented. It can be noticed that the sensor’s performance, derived from the optimization of the receptor layer, is competitive with other thermal platforms and devices based on electrochemical, microgravimetrical, and optical readout technologies.

**Table 2:** Comparison of Recently Developed Biomimetic Platform Applied to Food Samples

ref	sensor polymer/imprinting protocol/transducer	LoD CFU/mL	sample
(18)	polyurethane/microcontact/HTM	1 × 10^4^	buffer
(46)	poly(methyl methacrylate)/microcontact/SPR,QCM	1.5 × 10^6^, 3.7 × 10^5^	apple juice
(47)	polydopamine/electrodeposition^a^/electrochemical	8	water
(23)	polyurethane/microcontact/modified HTM	100	apple juice
(36)	polyurethane-*co*-urea/microcontact/HTM	1000	milk
present work	PDMS-GO/interfacial/HTM	70	strawberry-watermelon juice

a Electrodeposition: oxidation of conjugated monomers in order to form a conductive polymeric film.

## Conclusions

This work introduced a facile and novel bacteria-imprinting protocol for PDMS as a strategy to improve the analytical performance of the original heat transfer method sensing technology. This technique does not only attain a dense coverage of cavities on the receptors but also excludes the need for a template stamp. In combination with the use of a commercial resin, this procedure adds to the scalability of the synthesis process when compared with the state of the art imprinting techniques. Moreover, graphene oxide as an additive to the synthetic receptors has been investigated for the first time for the enhancement of the thermal transducer’s signal, massively decreasing the limit of detection for *Escherichia coli* to 80 ± 10 CFU/mL. This sensitivity is competitive to other thermal devices that have focused solely on the modification of the readout by implementing extra components, which require periodical calibration and add complexity to the device. The major advantage of the novel approach undertaken in this study is that the sensor’s performance is enhanced while the simple, low-cost, and user-friendly nature of the sensing technology is not sacrificed.

The performance of the proposed sensor was compared to a commercial impedance analyzer, obtaining similar sensitivity. Despite the fact that the developed receptors were assessed simultaneously for the two transducing technologies, the obtained data suggest that better results are expected when coupling the imprinted polymers to the separate readout platforms. Nonetheless, every sensitive electrochemical readout platform will benefit from some form of temperature control, while the sensor readout used in this case is not more complicated than the average temperature control unit in a commercial impedance analyzer.

Finally, the main objective of the research was to improve the technology for application in food safety management settings. The enhancement of the biomimetic thermal could enable end-users to faithfully determine the concentration of bacteria in commercial fruit juices within regulatory limits without any sample preparation. Further investigation of the performance of this device will be mainly focused on practical application of the sensor in hygiene monitoring of various food matrices. In order to do so, the desired performance of the sensor in light of the specific application at hand will have to be analyzed so that the PDMS can be chemically modified to tweak the selectivity of the layers to the desired level. Validation of the sensor in such a setting against the current standards of microbiological testing protocols will further illustrate the true potential of the sensor in food safety applications. In addition, the results summarized in this paper also illustrate that the concept could readily be extended toward application in other fields such as medical diagnostics or environmental screening.

## Supporting Information Available

Brightfield images and HTM results for the optimization of the sensing surface, scanning electron microscopy images of the PDMS-GO imprinted layers as well as the representative impedance/HTM simultaneous readout curves (PDF)

## ABBREVIATIONS

PDMS: polydimethylsiloxane
GO: graphene oxide
HTM: heat transfer method
SIP: surface imprinted polymer
NSIP: non-surface imprinted polymer
SEM: scanning electron microscope
R_th_: thermal resistance
PBS: phosphate buffer saline
LOD: limit of detection
QCM: quartz crystal microbalance
SPR: surface plasmon resonance
PID: proportional-integral-derivative
