# Supplementary material for: Imprinted Polydimethylsiloxane-Graphene Oxide Composite Receptor for the Biomimetic Thermal Sensing of Escherichia coli
Source: ACS Sens. 2022 May 10;7(5):1467–75. doi: 10.1021/acssensors.2c00215 (PMC9150177; doi:10.1021/acssensors.2c00215)
Supplement: Supplementary file 1 — se2c00215_si_001.pdf [file se2c00215_si_001.pdf]

Imprinted polydimethylsiloxane-graphene oxide composite receptor for the biomimetic thermal sensing of *Escherichia coli*.

-

Supplementary Information

-

Rocio Arreguin-Campos [a]*, Kasper Eersels [a], Renato Rogosic [a], Thomas J. Cleij [a], Hanne Diliën [a], Bart van Grinsven [a]

[a] Sensor Engineering Department, Faculty of Science and Engineering, Maastricht University, P.O.

Box 616, 6200 MD Maastricht, the Netherlands

*Corresponding author: r.arreguincampos@maastrichtuniversity.nl

Supplementary Figure 1

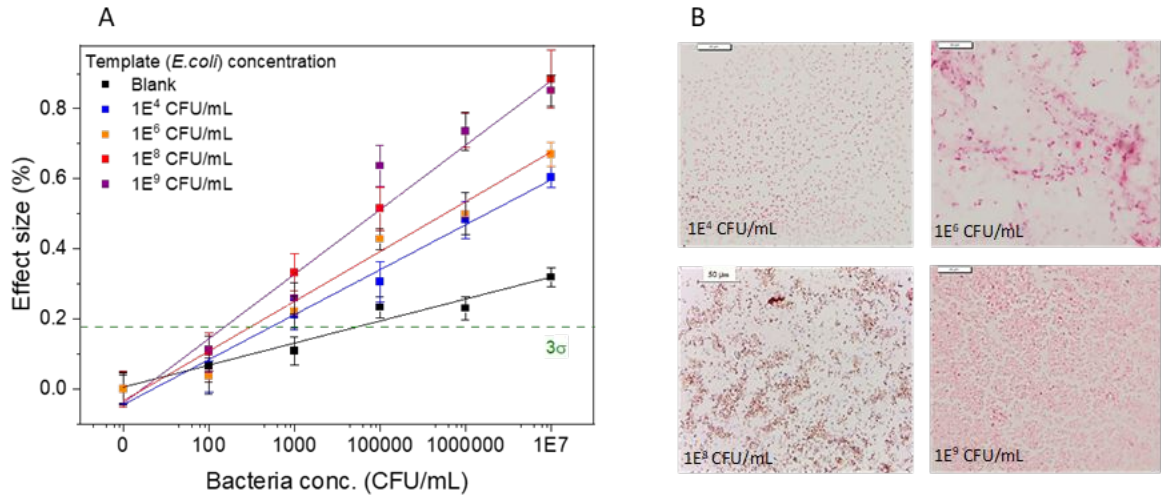

Optimization of the PDMS sensing surface. A) HTM real-time response comparison for the rebinding of bacteria towards imprints prepared using different concentrations of template for interfacial imprinting. B) Representative Brigthfield microscope images of the PDMS SIPs obtained by employing different template bacteria concentrations.

Supplementary Figure 2

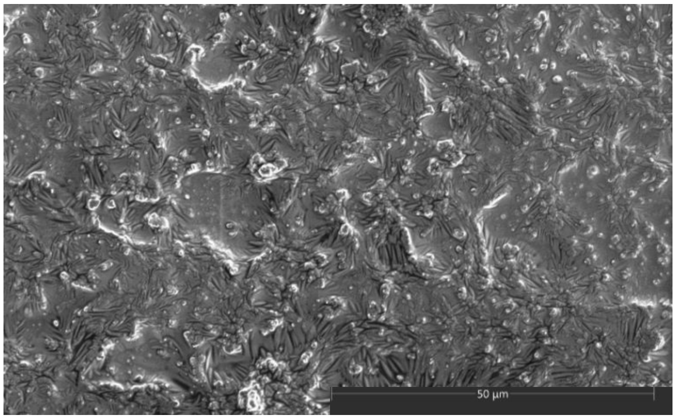

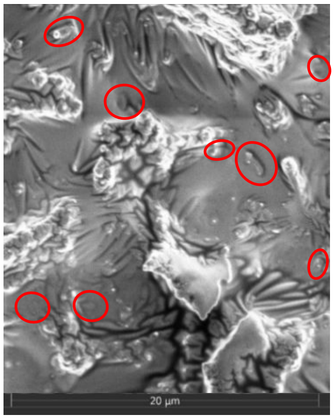

Scanning electron microscopy images of PDMS-GO imprinted layers. Red circles indicate bacteria imprints.

Supplementary Figure 3

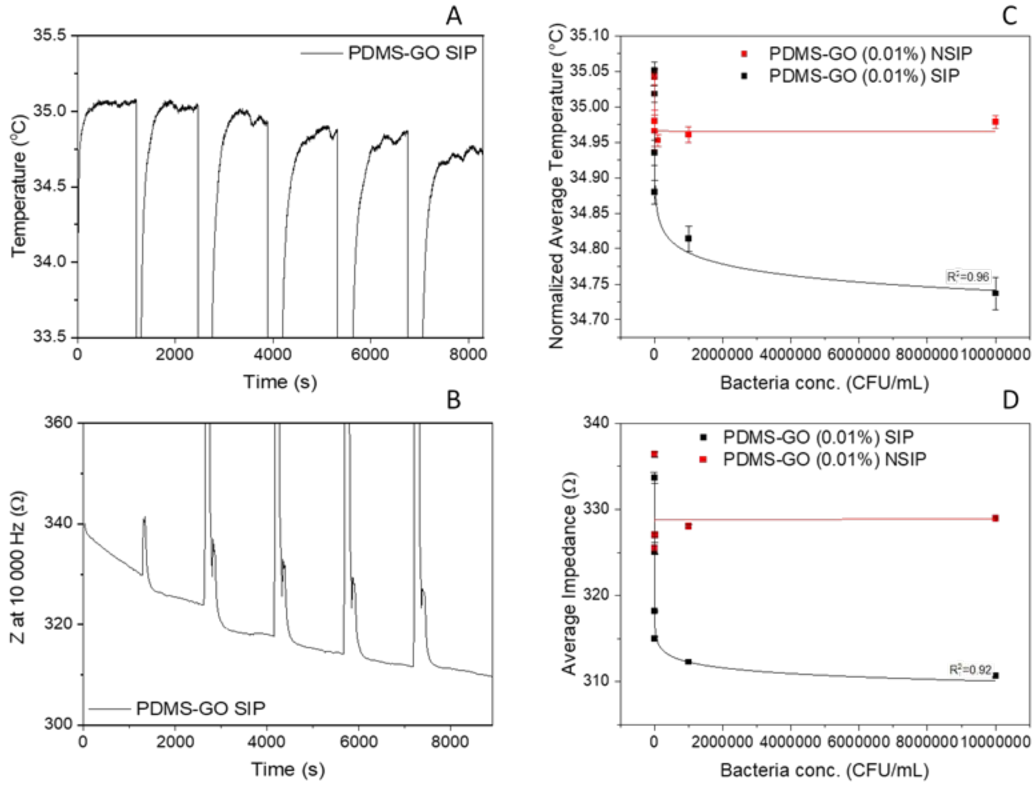

Simultaneous HTM (A) and impedance (B) real-time readout results for PDMS-GO receptor layer. Bacteria concentrations used were 0, $1E^2$, $1E^3$, $1E^4$, $1E^6$ and $1E^7$ CFU/mL. Dose-response fitted curves for the extracted temperature (C) and impedance (D) data.
